# Metabolic reconstruction of the near complete microbiome of the model sponge *Ianthella basta*

**DOI:** 10.1111/1462-2920.16302

**Published:** 2022-12-23

**Authors:** Joan Pamela Engelberts, Steven J. Robbins, Craig W. Herbold, Florian U. Moeller, Nico Jehmlich, Patrick W. Laffy, Michael Wagner, Nicole S. Webster

**Affiliations:** ^1^ Australian Centre for Ecogenomics, School of Chemistry and Molecular Biosciences The University of Queensland Brisbane Queensland Australia; ^2^ Centre for Microbiology and Environmental Systems Science, Division of Microbial Ecology University of Vienna Austria; ^3^ Department of Molecular Systems Biology Helmholtz‐Centre for Environmental Research – UFZ Leipzig Germany; ^4^ Australian Institute of Marine Science Townsville Queensland Australia; ^5^ Center for Microbial Communities, Department of Chemistry and Bioscience Aalborg University Aalborg Denmark; ^6^ Australian Antarctic Division Kingston Tasmania Australia

## Abstract

Many marine sponges host highly diverse microbiomes that contribute to various aspects of host health. Although the putative function of individual groups of sponge symbionts has been increasingly described, the extreme diversity has generally precluded in‐depth characterization of entire microbiomes, including identification of syntrophic partnerships. The Indo‐Pacific sponge *Ianthella basta* is emerging as a model organism for symbiosis research, hosting only three dominant symbionts: a Thaumarchaeotum, a Gammaproteobacterium, and an Alphaproteobacterium and a range of other low abundance or transitory taxa. Here, we retrieved metagenome assembled genomes (MAGs) representing >90% of *I. basta*'s microbial community, facilitating the metabolic reconstruction of the sponge's near complete microbiome. Through this analysis, we identified metabolic complementarity between microbes, including vitamin sharing, described the importance of low abundance symbionts, and characterized a novel microbe–host attachment mechanism in the Alphaproteobacterium. We further identified putative viral sequences, highlighting the role viruses can play in maintaining symbioses in *I. basta* through the horizontal transfer of eukaryotic‐like proteins, and complemented this data with metaproteomics to identify active metabolic pathways in bacteria, archaea, and viruses. This data provide the framework to adopt *I. basta* as a model organism for studying host–microbe interactions and provide a basis for in‐depth physiological experiments.

## INTRODUCTION

Sponges are ecologically important components of aquatic ecosystems around the world, providing habitat and mediating biochemical cycles by filtering thousands of litres of water each day (Bell, 2008; de Goeij et al., 2013; Weisz et al., 2008). On coral reefs, for example, sponges recycle and retain nutrients through the ‘sponge‐loop’, in which sponges take up and transform dissolved organic matter (DOM) into biomass, which in turn is consumed by reef‐dwelling detritivores to complete the loop (de Goeij et al., 2013). Marine sponges host diverse and stable communities of microorganisms that are distinct from the surrounding seawater (Schmitt et al., 2012; Taylor et al., 2013) and contribute to holobiont health and overall function. For example, sponge symbionts are actively involved in the assimilation of DOM, recycle host waste products, metabolize toxic ammonia, and are enriched in restriction‐modification systems that could aid in defence against mobile genetic elements, such as viruses (Hudspith et al., 2021; Moeller et al., 2019; Pita et al., 2018; Robbins et al., 2021; Slaby et al., 2017).

Despite the importance of the sponge microbiome, its complexity hampers the in‐depth characterization of each microbial taxa, with many sponge species hosting up to 3000 distinct microbial species (Webster et al., 2010). Consequently, few sponge symbionts have been described in detail and hypotheses on microbial syntrophy have remained confined to select members of the community (Bayer et al., 2020; Moitinho‐Silva et al., 2017). Moreover, sponge species for which the function of all dominant microbial members has been inferred from omics data frequently lack data on symbionts present in low abundance (Gauthier et al., 2016). However, low abundance microbes can drive fundamentally different processes than high abundance microbes (Rivett & Bell, 2018). To systematically understand how the sponge holobiont functions and to allow for manipulative experiments to further unravel the mechanisms of establishment and maintenance of symbiosis, fully characterized model organisms are needed. Such models will also be extremely valuable for studying more complex animal–microbiota symbiotic relationships.

*Ianthella basta* is an abundant sponge throughout the Indo‐Pacific and hosts only three dominant symbionts: a Thaumarchaeotum, a Gammaproteobacterium, and an Alphaproteobacterium (Luter et al., 2010; Moeller et al., 2019), and a range of other low abundance taxa, making this sponge an ideal candidate for establishing a model for sponge symbiosis. *Ianthella basta*'s microbiome is stable across different environments (Luter et al., 2010), with the dominant symbionts, which are vertically transmitted (Engelberts et al., 2022), typically comprising over 90% of the community. Previous metagenomic and physiological studies of *I. basta* have characterized the dominant Thaumarchaeotum and Gammaproteobacterium as key players within important pathways, such as ammonia oxidation and taurine metabolism, respectively (Moeller et al., 2019, 2022). Here, we complete the microbiome by recovering MAGs for the dominant Alphaproteobacterium and several low abundant microbes that are persistent across samples, which allowed us to not only describe the full genomically predictable metabolic potential of *I. basta*'s microbiome, but also to reconstruct metabolic complementarity between these microbes. We further highlight the contribution of viruses to the metabolic capability of the sponge and complement all data with metaproteomic sequencing. This study provides an unprecedented framework within which to use *I. basta* in future manipulative experiments (Engelberts et al., 2021).

## EXPERIMENTAL PROCEDURES

### Sample collection

The sponge *Ianthella basta* presents in two colour morphotypes, purple and yellow (Freckelton et al., 2012), of which the tissue can be designated as either thick or thin. For this study, three yellow *Ianthella basta* individuals with thin tissue were collected in November 2019 from Orpheus Island, central Great Barrier Reef (18°37′ S, 146°30′ E) between 11 and 15 m depth using scuba. Sponges were kept in aquaria for 12 h, after which they were fragmented, snap frozen, and sent to the Australian Centre for Ecogenomics (ACE), Brisbane, for metagenomic sequencing and to the Centre for Environmental Research, Helmholtz (Leipzig, Germany), for metaproteomic analysis.

To determine whether the metagenomes recovered in this study were representative of the dominant symbionts found in different morphotypes of *I. basta*, we blasted previously recovered 16S rRNA gene amplicon sequences of the three dominant symbionts of a purple, thick morphotype (Engelberts et al., 2022) to our genomes using Blast v2.9.0+ (Camacho et al., 2009). A further three yellow, thin and three yellow, thick individuals were collected from Davies Reef (S 18°49.354′, E 147°38.253′) in February 2021, using SCUBA at depths between 12 and 15 m depth. For these individuals, 16S rRNA gene amplicon sequences of the three dominant symbionts were obtained following the methods described in Engelberts et al. (2022) and again blasted to the genomes from this study (see Table S2 for 16S rRNA gene amplicon sequences).

### Microbial enrichment, DNA extraction, and metagenomic sequencing

Microbial cells were separated from the host tissue for all three *I. basta* individuals (~1 to 3 g sponge wet weight per individual) according to the methods described in Botté et al. (2019), with the exception that samples were ground on ice in 1X Calcium/Magnesium‐Free Sea Water (CMFSW) for 5 min to homogenize the sponge tissue. Samples were treated with DNase I (RNase‐free, New England Biolabs, M0303) to remove any remaining host and extracellular DNA, after which the microbial DNA was extracted using the phenol‐chloroform protocol described in Robbins et al. (2019), omitting all freeze–thaw cycles which could shear the DNA. We note that it is possible that the removal of host cells could remove intracellular symbionts, such as Chlamydiae (Engelberts et al., 2022). Quantity and quality of the extracted DNA were checked using Qubit assays and 16S/18S rRNA PCR. Libraries were prepared with the Illumina Nextera Flex library prep kit at Microba Life Sciences (Brisbane, Australia) and sequenced on the Illumina NovaSeq 6000 (2 × 250 bp). All samples (*n* = 3) were first sequenced at an average depth of 5.5 Gbp for differential coverage binning (Albertsen et al., 2013), followed by sequencing at a depth of 20 Gbp to increase the coverage of microorganisms present at low abundance.

### Metagenome assembly, binning, and taxonomic assignment

Paired‐end DNA sequencing data were demultiplexed and adapters trimmed using Illumina BaseSpace Bcl2fastq2 v2.20, accepting one mismatch in index sequences. All reads were assembled using metaSPades v3.14.0 (Nurk et al., 2017). To supplement this dataset, microbial metagenomic reads previously retrieved from six sequencing runs on one purple, thick *I. basta* individual (sampled in October 2011; Moeller et al., 2019) were reassembled using metaSPAdes v3.14.0 (Nurk et al., 2017) and analysed together with data from this study. Binning of all data (separately for each sponge individual) was performed with the ‘recover’ pipeline of Aviary (https://github.com/rhysnewell/aviary), which uses the binning algorithms MetaBAT v1 (Kang et al., 2015), MetaBAT v2 (Kang et al., 2019), MaxBin v2 (Wu et al., 2015), CONCOCT (Alneberg et al., 2014), VAMB (Nissen et al., 2021), and Rosella (https://github.com/rhysnewell/rosella). DASTool v1.1.2 (Sieber et al., 2018) was used to select for the best representative metagenome assembled genome (MAG) for each sponge individual produced from the different binning algorithms. Completeness and contamination of each MAG were determined using CheckM v1.1.3 (Parks et al., 2015) and only MAGs that were over 50% complete with less than 5% contamination were retained for downstream analyses.

GTDB‐Tk v1.5.0 (Chaumeil et al., 2019), which is based on the Genome Taxonomy Database (GTDB, http://gtdb.ecogenomic.org) taxonomy Release 202, was used to assign taxonomy to each MAG. GTDB‐Tk uses FASTANI (Jain et al., 2018) and pplacer (Matsen et al., 2010) to classify MAGs based on their placement in a reference tree, which is inferred using a set of 120 bacterial and 122 archaeal markers.

**FIGURE 1:**
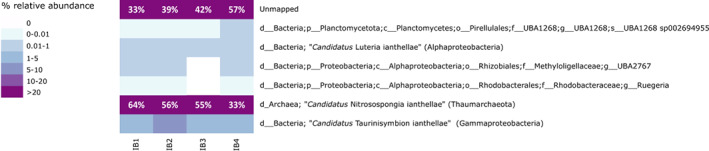
Relative abundance of MAGs present in ≥3 *I. basta* individuals after de‐replication at 95% identity. For *I. basta* individual 4 the average relative abundance is shown, as this individual was sequenced six times in the study Moeller et al. (2019). ‘Unmapped’ represents reads that could not be mapped to any of the six MAGs. IB: *Ianthella basta* followed by the replicate number. The proposed species name (dominant symbionts; Moeller et al., 2019, 2022) or the GTDB‐Tk taxonomy (low abundant symbionts) is displayed.

MAGs were dereplicated at 95% identity with Drep v2.6.2 using otherwise default parameters (Olm et al., 2017) to remove duplicate MAGs recovered from *I. basta* individuals from our study and Moeller et al. (2019). To confirm that the recovered MAGs represented the majority of *I. basta*'s microbial community, single‐copy marker genes were identified in all raw reads and MAGs and the fraction of marker genes captured in the MAGs was subsequently assessed using the ‘pipe’ and ‘appraise’ functions of SingleM v0.13.2 (https://github.com/wwood/singlem). To calculate the relative abundance of each MAG in all four *I. basta* individuals (three from this study, one study from Moeller et al., 2019), MAGs were first dereplicated at 95% identity with Drep v2.6.2 (Olm et al., 2017) to avoid arbitrary mapping between representatives of highly similar genomes, and reads were mapped to these MAGs with CoverM v0.2.0 (https://github.com/wwood/CoverM, min‐covered‐fraction set to 0.1). The final heatmap was visualized with R v4.0.5 (R Core Team, 2013), showing reads from the deep sequencing runs on the three *I. basta* individuals from this study, reads from one *I. basta* individual from Moeller et al. (2019), and MAGs that were consistently present over the four *I. basta* individuals (i.e. present in at least three out of the four individuals; Figure 1). These MAGs were used for the metabolic reconstruction of *I. basta*'s microbiome. We further analysed a dataset originating from a 454‐pyrosequencing run from Moeller et al. (2019). From this dataset, a Thaumarchaeotal and Gammaproteobacterial MAG were retrieved with ANI values of 98.90% and 97.78% compared to the genomes recovered in this study. However, due to the low sequencing depth, this data was not included in Figure 1.

### Tree building

Archaeal and bacterial phylogenomic trees were inferred for each of *I. basta*'s three dominant symbionts, using the ‘de_novo_wf’ workflow of GTDB‐Tk v1.5.0 (Chaumeil et al., 2019). This workflow calculates de novo trees using FastTree v2.1.9 (Price et al., 2010) using the WAG + GAMMA model. Each tree included all publicly available genomes from GTDB that belonged to the same phylum or class as the respective *I. basta* symbiont ‐ i.e. all Thaumarchaeota (a total of 564 MAGs), Gammaproteobacteria (8980 MAGs), or Alphaproteobacteria (7361 MAGs). Tree files were further visualized and refined with the Interactive Tree Of Life (iTOL) v6.4.1 (Letunic & Bork, 2021) and Inkscape v0.92.4 (https://inkscape.org/) for Figures [Link], [Link].

The closest relatives to the *I. basta* Thaumarchaeotal MAG and Gammaproteobacterial 16S rRNA gene amplicon sequence (see Moeller et al., 2022) came from the sponge *Hexadella detritifera*, which like *I. basta*, belongs to the *Ianthellidae* family. To elucidate whether the dominant Alphaproteobacterial and Gammaproteobacterial MAGs from *I. basta* were also most closely related to microbial symbionts found in *H. detritifera*, we assembled and binned previously published raw reads from *H. detritifera* (Zhang et al., 2019). The resulting Alpha‐ and Gammaproteobacterial MAGs were added to the phylogenomic trees (see Supplementary Note 1).

### Functional annotation of MAGs

The EnrichM v0.6.3 (https://github.com/geronimp/enrichM) ‘annotate’ function was used to functionally annotate MAGs with the Kyoto Encyclopaedia of Genes and Genomes (KEGG) Orthologies (KOs) and PFAM databases (Kanehisa et al., 2015; Mistry et al., 2020), as well as with Hidden Markov Models (HMMs) from the database for Automated carbohydrate‐Active Enzyme Annotation (dbCAN) (Yin et al., 2012) to identify carbohydrate‐active enzymes (CAZYs). For carbon fixation, vitamin production, and amino acid biosynthesis pathways, EnrichM v0.6.3 ‘classify’ was used to identify pathways that were ≥60% complete and consequently assigned to a MAG. Notably, the KEGG module for cobalamin biosynthesis does not include the full pathway, thus the cobalamin module defined by Engelberts et al. (2020) was used. The presence of amino acid biosynthesis pathways was further validated using GapMind (Price et al., 2020). For ABC transporters to be positively assigned to a MAG, genes for both the substrate‐specific component and the integral membrane protein had to be present. Secondary metabolite clusters were predicted in each MAG using AntiSMASH v5.0 (Blin et al., 2019). Proteins with predicted signal peptides for the translocation across the cytoplasmic and outer membrane were identified using SignalP v5.0 (Almagro Armenteros et al., 2019).

### Identification, cross‐validation, and annotation of viral sequences

Putative viral sequences were identified in all metagenomic assemblies using VirSorter v1.1 (Roux et al., 2015). To assign taxonomy, the putative viral sequences were aligned to the viral component of the RefSeq Nonredundant (Nr) database (https://www.ncbi.nlm.nih.gov/refseq/), with an E‐value cut‐off of 1e‐05. Sequences were also blasted against previously recovered viral sequences from *I. basta* (Laffy et al., 2018) using BlastP v2.9.0 (Camacho et al., 2009) for cross‐validation and to further confirm their taxonomic assignment (Table S6). Viral contigs were functionally annotated by calling open reading frames (ORFs) in the metagenomic assemblies using Prodigal v2.6.3 (Hyatt et al., 2010). ORFs were subsequently annotated with the KO and PFAM databases using EnrichM v0.6.3 (https://github.com/geronimp/enrichM).

### Protein extraction and proteome analyses

Tissue from the same three *I. basta* individuals metagenomically sequenced in this study was sent to the Centre for Environmental Research, Helmholtz, for metaproteomic analysis. Sponge tissue was disrupted by bead beating (FastPrep‐24, MP Biomedicals, Sanra Ana, CA, USA; 5.5 ms, 1 min, 3 cycles) followed by ultra‐sonication (UP50H, Hielscher, Teltow, Germany; cycle 0.5, amplitude 60%) and centrifugation (10,000 × *g*, 10 min). The protein lysate was loaded on an SDS‐gel and run for 10 min. The gel piece was cut, washed, and incubated with 25 mM 1,4 dithiothreitol (in 20 mM ammonium bicarbonate) for 1 h and 100 mM iodoacetamide (in 20 mM ammonium bicarbonate) for 30 min, and destained, dehydrated, and proteolytically cleaved overnight at 37°C with trypsin (Promega). Digested peptides were extracted and desalted using ZipTip μC18 tips (Merck Millipore, Darmstadt, Germany). Peptide lysates were re‐suspended in 15 μl 0.1% formic acid and analysed by nanoliquid chromatography mass spectrometry (UltiMate 3000 RSLCnano, Dionex, Thermo Fisher Scientific). Mass spectrometric analyses of eluted peptide lysates were performed on a Q Exactive HF mass spectrometer (Thermo Fisher Scientific) coupled with a TriVersa NanoMate (Advion, Ltd., Harlow, UK). Peptide lysates were injected on a trapping column (Acclaim PepMap 100 C18, 3 μm, nanoViper, 75 μm × 2 cm, Thermo Fisher Scientific) with 5 μl/min by using 98% water/2% ACN 0.5% trifluoroacetic acid and separated on an analytical column (Acclaim PepMap 100 C18, 3 μm, nanoViper, 75 μm × 25 cm, Thermo Fisher Scientific) with a flow rate of 300 nl/min. Mobile phase was 0.1% formic acid in water (A) and 80% ACN/0.08% formic acid in water (B). Full MS spectra (350–1550 m/z) were acquired in the Orbitrap at a resolution of 120,000 with automatic gain control (AGC) target value of 3 × 106 ions.

Data resulting from LC–MS/MS measurements were analysed with the Proteome Discoverer v2.4 (Thermo Fischer Scientific) using SEQUEST HT. Protein identification was performed using a custom protein database, which consisted of proteins identified by Prodigal v2.6.3 (Hyatt et al., 2010) in all metagenomic assemblies and MAGs from the three *I. basta* individuals from this study and the one from Moeller et al. (2019). All protein sequences were functionally annotated with the KO and PFAM databases using EnrichM v0.6.3 (https://github.com/geronimp/enrichM). To avoid arbitrary mapping, only non‐redundant protein sequences were used for protein identification (clustered at an identity threshold of 100%). Searches were conducted with the following parameters: Trypsin as enzyme specificity and two missed cleavages allowed. A peptide ion tolerance of 10 ppm and an MS/MS tolerance of 0.02 Da were used. As modifications, oxidation (methionine) and carbamidomethylation (cysteine) were selected. Peptides that scored a q‐value >1% based on a decoy database and with a peptide rank of 1, were considered identified. Redundant proteins were grouped in protein groups by applying the strict parsimony principle. Only the protein groups that explain at least one unique identified peptide were reported through the Top3 approach implemented in the Proteome Discoverer v2.4.

This protein dataset was supplemented with previously recovered metaproteomics data from the *I. basta* individual sampled in Moeller et al. (2019). To this end, all 49 raw protein spectra from Moeller et al. (2019) (https://www.ebi.ac.uk/pride/archive/projects/PXD012484) were searched against the custom *I. basta* protein database using the same parameters as described above. However, as the MS‐analysis in Moeller et al. (2019) was performed on a LTQ Velos Orbitrap and MS/MS spectra were measured in ‘unit resolution’‐mode, protein reports were further filtered so that each listed protein had at least two peptides to prevent the identification of a protein based on one peptide.

## RESULTS AND DISCUSSION

### General MAG statistics and selection

In total, 43 microbial MAGs were recovered from four *I. basta* individuals, of which 23 originated from the three *I. basta* individuals collected in this study and 20 from an individual sampled in 2011 (Moeller et al., 2019). The recovered MAGs had an average completeness of 86% ± 13%, contamination of 1% ± 1%, and GC content of 61% ± 3% (Table S1). In contrast to the low GC percentage typically observed in seawater microbes (Giovannoni et al., 2005), a relatively high GC content (i.e. >50%) is commonly observed in sponge symbiont metagenomes (Horn et al., 2016; Moeller et al., 2019). This could be driven by a high environmental availability of nitrogen (Luo et al., 2015) derived from the concentration of nutrients (DOM and DON) and the release of taurine by the sponge host (Moeller et al., 2022; Rix et al., 2020; Robbins et al., 2021). De‐replication of the MAGs at 95% identity to remove duplicate MAGs between *I. basta* individuals reduced the total number to 10, including one archaeal MAG from the phylum Thaumarchaeota representing the previously characterized ‘*Candidatus* Nitrosospongia ianthellae*'* (Moeller et al., 2019) (Thermoproteota as per GTDB Release 202) and nine bacterial MAGs, belonging to the phyla Proteobacteria (classes Alpha‐ and Gammaproteobacteria; three MAGs and one MAG, respectively, the gammaproteobacterial MAG represents the previously analysed ‘*Candidatus* Taurinisymbion ianthellae’; Moeller et al., 2022), Planctomycetota (three MAGs), Cyanobacteria (1), and Acidobacteriota (1). All genomes of the three dominant symbionts recovered from the four *I. basta* individuals had an ANI between 96.1% and 99.9%. Analysis of the relative abundance of the MAGs in each *I. basta* individual showed that MAGs belonging to the phyla Acidobacteriota, Cyanobacteria, and Planctomycetetota (two MAGs) were absent in half of the individuals. Thus, these MAGs were omitted from further analyses. The resulting six MAGs that were consistently (i.e. in ≥3 of the 4 tested individuals) present across *I. basta* individuals had a relative abundance between 0.2% ± 0.6% and 51% ± 11% (Figure 1). Based on the presence of single‐copy marker genes identified by SingleM in both the raw reads and MAGs, these six symbionts represented 92% of the sponge's microbial community, allowing for the metabolic reconstruction of all major members of the *I. basta* microbiome (i.e. the near complete microbiome). The remaining 8% of the detected microbial community likely represent food bacteria or very low abundance symbionts.

### Dominant symbionts fall within sponge‐specific symbiont clades

To determine whether metagenomes recovered in this study were representative of the dominant symbionts found in all morphotypes of *I. basta*, we blasted previously (purple, thick morphotype) and newly (yellow, thin and thick morphotype) recovered 16S rRNA gene amplicon sequences of the three dominant symbionts to the genomes from this study (yellow, thin morphotype). All 16S rRNA gene sequences mapped with ≥99.2% identity to their respective genome, showing that highly similar symbiont communities are found in geographically and morphologically different *I. basta* individuals (Table S2). To then determine the phylogenetic placement and closest relatives of *I. basta*'s dominant symbionts, three phylogenomic trees were inferred for the Thaumarchaeotum ‘*Candidatus* Nitrosospongia ianthellae’ (Figure S1), the Gammaproteobacterium ‘*Candidatus* Taurinisymbion ianthellae’ (Figure S2), and the abundant Alphaproteobacterium (Figure S3). All three dominant symbionts clustered with previously recovered microbial MAGs from sponges, allowing for the extrapolation of our findings to other sponge species and supporting the wider applicability of *I. basta* as a model species for symbiosis research. The Thaumarchaeotum ('*Ca*. Nitrosospongia ianthellae'; Moeller et al., 2019), which was the most abundant symbiont in *I. basta* (Figure 1), was most closely related to MAGs from the genus Nitrosopumilus, associated with the sponge *Hexadella dedritifera* (Zhang et al., 2019) (64.00% average amino acid identity [AAI] to GB_GCA_003724325.1; Figure S1), which, like *I. basta*, is a member of the sponge family *Ianthellidae*. '*Ca*. Nitrosospongia ianthellae' also clustered with Nitrosopumilus‐related MAGs from the marine demosponge *Coelocarteria singaporensis* (Botté et al., 2019). *Ianthella basta'*s dominant Gammaproteobacterium (‘*Ca*. Taurinisymbion ianthellae’; Moeller et al., 2022) was most closely related to two MAGs from the LS‐SOB family which were recovered from the sponge *H. dedritifera* (79.83% AAI to Hexadella_Gamma_MAG_1 and 77.48% AAI to Hexadella_Gamma_MAG_2, see Supplementary Note 1). Together, these three genomes formed a sister group to one LS‐SOB MAG from the sponge *Ircinia ramosa* (Engelberts et al., 2020), which in turn formed a sister group to six other LS‐SOB MAGs from the sponges *Coscinoderma matthewsi* (Glasl et al., 2020), *Lophophysema eversa* (Tian et al., 2016), *Mycale hentscheli* (Rust et al., 2020; Storey et al., 2020), and *Ircinia ramosa* (Engelberts et al., 2020) (Figure S2). The Alphaproteobacterium (o_JABSOH01) had the lowest resolved taxonomy of the three dominant symbionts and was most closely related to an Alphaproteobacterial MAG recovered from *H. dedritifera* (71.68% AAI), which together formed a sister group to six genomes found in the deep‐sea sponge *Vazella pourtalesii* (Bayer et al., 2020) (Figure S3) belonging to the same order. Compared to the six Alphaproteobacterial MAGs from the deep‐sea sponge *Vazella pourtalesii*, the Alphaproteobacterial MAG in *I. basta* had highest AAI of 43.87% with GCA_014238935.1 (Figure S3), which was well below an AAI of 60%–80% that is typical for MAGs grouped at the same genus (Konstantinidis et al., 2017). Thus, we propose that this symbiont represents a new species within a new genus and tentatively named it ‘*Ca*. Luteria ianthellae’. The species name ianthellae refers to the host of this microorganism, the sponge *Ianthella basta*. The genus is named after Dr. Heidi Luter, who performed the foundational work on *I. basta*.

All three dominant symbionts of *I. basta* were most closely related to microbial symbionts of *H. dedritifera*. Both sponge species are members of the *Ianthellidae* family, suggesting that the three main symbionts were acquired by a common ancestor of these two sponge genera. Future research should focus on characterizing the microbial communities of other members of this sponge family to better understand the evolutionary history of the *Ianthellidae* microbiome.

### General overview of metaproteomics data

In total, 11,543 expressed protein sequences (8675 non‐redundant) were recovered from the four *I. basta* individuals, (where three were sequenced in this study and one in the study by Moeller et al., 2019), of which 6480 could be functionally annotated, either by the (KEGG) Orthologies (KOs) or PFAM database (Table S3). A total of 1700 proteins were captured in the genomes of *I. basta*'s six symbionts of which 1134 were functionally annotated: 631 (37%) protein sequences were assigned to the Gammaproteobacterium, 496 (29%) to the dominant Alphaproteobacterium, 354 (21%) to the Thaumarchaeotum, 124 (7%) to Ruegeria (Alphaproteobacterium), 88 (5%) to UBA1268 (Planctomycetota), and 7 (0.4%) to UBA2767 (Alphaproteobacterium; Table S3).

### Metaproteogenomic analysis of *Ianthella basta's* microbiome

**FIGURE 2:**
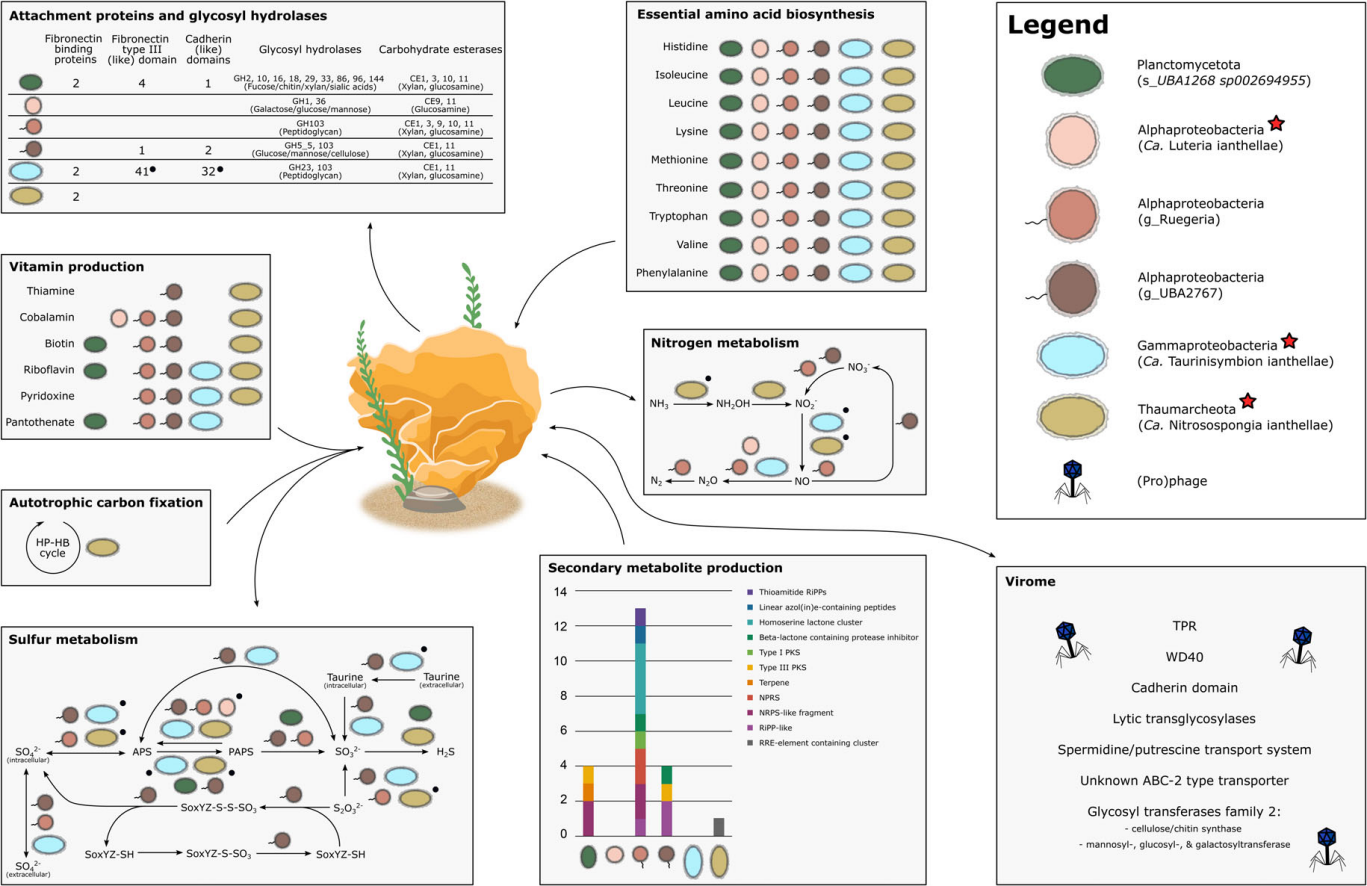
Metabolic reconstruction of *I. basta*'s microbiome and virome. Arrowheads point to the hypothesized direction of transfer. Genes expressed in the metaproteomics data are indicated by a black dot. For secondary metabolite production and vitamin and amino acid biosynthesis, proteins were either not detected (secondary metabolite production) or detected for <60% of the genes in the metabolic pathway (vitamin and amino acid biosynthesis). The legend displays the proposed species name (for the dominant symbionts, Moeller et al., 2019, 2022) or the lowest resolved taxonomy as per GTDB taxonomy Release 202, with the three dominant symbionts highlighted by a star. HP‐HB cycle, hydroxypropionate‐hydroxybutyrate cycle; NPRS, non‐ribosomal peptide synthetase; PKS, polyketide synthase; RiPP, ribosomally synthesized and post‐translationally modified peptides; RRE, RiPP recognition element; TPR, tetratricopeptide repeats

A metaproteogenomic analysis of *I. basta*'s complete microbiome provided unique insights into the distribution of functions across the six symbionts (Figure 2, Tables S3 and S4). For example, microbial oxidation of ammonia can prevent its accumulation to toxic levels (Zhang et al., 2014), benefitting the sponge host. Functional analysis of the gene responsible for this step, ammonia monooxygenases (*amoA*), revealed that only Thaumarchaeota are capable of ammonia oxidation in sponges (Robbins et al., 2021), which was confirmed in *I. basta* with isotope‐based functional assays (Moeller et al., 2019), and further corroborated here (Figure 2). Interestingly, *I. basta* harbours no nitrite oxidizer and thus performs as a holobiont incomplete nitrification of ammonia to nitrite (Moeller et al., 2019). In addition, we show that low abundance microbes have the genomic potential to reduce nitrite and nitric oxide produced by the oxidation of ammonia. For example, one of the two low abundant Alphaproteobacteria (g_Ruegeria) encodes genes for the reduction of nitrite to dinitrogen through nitric and nitrous oxide (i.e. denitrification, catalysed by *nirS*, *norBC*, and *nosZ*), which could potentially release nitrogen from the system (Figure 2, Table S4). Notably, the first two steps of denitrification can also be performed by the Gammaproteobacterium (‘*Ca*. Taurinisymbion ianthellae’) through NirK and qNOR, of which the latter was further encoded in '*Ca*. Luteria ianthellae'. Alternatively, nitric oxide could be detoxified to nitrate via the other low abundance alphaproteobacterium (g_UBA2767), catalysed by nitric oxide dioxygenase (Hmp; Figure 2, Table S4). Interestingly, denitrification and nitrate production were previously suggested to occur in *I. basta* based on an isotope‐based functional assays (Moeller et al., 2019), but the identity of the organisms responsible could not be confirmed. Based on our results, we suggest that the low abundance Alphaproteobacteria from the genera Ruegeria and UBA2767 are responsible for these key processes of removing and transforming nitrogen compounds from the sponge host.

Taurine is a naturally occurring compound in marine sponges, with concentrations up to 6 μmol/g wet weight measured in *I. basta* (Moeller et al., 2022). It has been shown that the gammaproteobacterium ‘*Candidatus* Taurinisymbion ianthellae’ has the metabolic potential to use taurine for energy conservation coupled with the release of sulfate and ammonia (Moeller et al., 2022). Interestingly, not only the abundant symbiont ‘*Ca*. Taurinisymbion ianthellae’ but also the low abundance Alphaproteobacterium (g_UBA2767) is well equipped for the use of taurine. The latter symbiont encodes the taurine transporter (*tauABC*) as well as genes for two taurine degradation pathways to convert taurine to sulfite (either via taurine dioxygenase [*tauD*] or taurine dehydrogenase [of which the large subunit *TauY* is encoded, but not the small subunit *TauX*] and sulfoacetaldehyde acetyltransferase [*xsc*]). This symbiont further encodes genes to subsequently convert sulfite into adenylyl sulfate (APS), via adenylylsulfate reductase (*aprAB*), and to reduce APS into 3′‐phosphoadenylyl sulfate (PAPS) via the bifunctional enzyme CysN/CysC (*cysNC*; Figure 2, Table S4). *AprAB* can further shuttle electrons from the oxidation of sulfite to the quinone pool. PAPS is an active sulfate donor, which can be used by microorganisms to synthesize sulfated compounds. PAPS can also be converted into sulfide via sulfite and incorporated into amino acids, such as cysteine. These reactions are catalysed by phosphoadenosine phosphosulfate reductase (*cysH*) and sulfite reductase (*sir*), respectively, of which the latter was found in the genomes of the Planctomycetota (g_UBA1268) and '*Ca*. Nitrosospongia ianthellae' (Figure 2). Alternatively, APS can be converted to sulfate via sulfate adenylyltransferase (*sat*), generating ATP, thus allowing the low abundance Alphaproteobacterium (g_UBA2767) to use taurine for energy conservation. Moreover, two of *I. basta*'s symbionts, the Alphaproteobacteria (g_UBA2767 and g_Ruegeria), are postulated to import sulfate from the surrounding seawater via sulfate permease (*sulP*). This environmentally obtained sulfate could also be reduced to PAPS for assimilation (Figure 2). However, it must be noted that sulfate permease can also export sulfate generated from taurine, which has been previously proposed to occur in '*Ca*. Taurinisymbion ianthellae' (Moeller et al., 2022). Overall, these findings suggest that sulfur metabolism in *I. basta*'s microbiome is predominantly geared towards gaining energy for the Alphaproteobacterium (g_UBA2767) and '*Ca*. Taurinisymbion ianthellae' and towards gaining cellular sulfur for the other symbionts. Notably, the Alphaproteobacterium (g_UBA2767) is predicted to also be able to obtain energy through the oxidation of thiosulfate to sulfate through sulfur‐oxidizing proteins (*soxAB*, *soxXYZ*), called the SOX complex (Tian et al., 2014). This further indicates the importance of the Alphaproteobacterium (g_UBA2767) in the sulfur metabolism of *I. basta*, which was initially thought to be solely carried out by the dominant Gammaproteobacterium, which can also convert DMSP (Moeller et al., 2022).

The third low abundance symbiont, the Planctomycetota (g_UBA1268), is not predicted to be a major player in nitrogen and sulfur metabolism, but it plays an important role in catabolizing glycoconjugates and carbohydrates via glycosyl hydrolases (GHs) and carbohydrate esterases (CEs; Figure 2). Marine sponges continuously (re)cycle dissolved organic matter (DOM), a rich source of complex sugars, and a recent study showed that sponge symbionts are actively involved in utilizing this DOM (Campana et al., 2021). Similarly, free‐living Planctomycetota play an important role in metabolizing complex carbohydrates (Martinez‐Garcia et al., 2012; Orellana et al., 2022; Sichert et al., 2020) Here, we found that *I. basta*'s Planctomycetota (g_UBA1268) encoded nine glycosyl hydrolases, acting amongst others on fucose (GH29), chitin (GH18), xylan (GH10), and sialic acids (GH33; Figure 2), compounds often found in marine algae or the sponge itself (Hsieh & Harris, 2019; Kappelmann et al., 2019; Morya et al., 2012; Mutsenko et al., 2017). These nine GHs were absent from all other symbionts, highlighting the unique role of the Planctomycetota (g_UBA1268) in breaking down a wide range of complex carbohydrates. Interestingly, the three Alphaproteobacterial symbionts and '*Ca*. Taurinisymbion ianthellae' could import sialic acid through the sialic acid tripartite ATP‐independent periplasmic transporter (*SiaPQM*). GH29 and GH33 were also expressed in the metaproteome of *I. basta*'s microbiome and have been identified previously as important hydrolase classes for many sponge symbionts and were often present in other sponge‐associated Planctomycetota (Robbins et al., 2021). The carbohydrate esterases that were identified in the genomes of *I. basta*'s symbionts predominantly targeted xylan (CE1) and glycosamine (CE11), a structural component of sponges (Fernandez‐Busquets & Burger, 2003; Kamke et al., 2013), while CE10, an arylesterase, was further encoded and expressed in the proteome of the Planctomycetota (g_UBA1268; Figure 2, Table S3). Arylesterases are involved in detoxifying reactive oxygen species, acting as antioxidants in humans (Asare et al., 2018), and could potentially play similar roles in marine sponges. No GHs and CEs were identified in the Thaumarchaeotum, which was consistent with previous broad‐scale analyses on sponge associated Thaumarchaeota (Robbins et al., 2021). Notably, Thaumarchaeota are predominantly autotrophic and primarily obtain their carbon from fixation via the 3‐hydroxypropionate/4‐hydroxybutyrate cycle (Figure 2) (Burgsdorf et al., 2021; Engelberts et al., 2020; Moeller et al., 2019; Offre et al., 2013; Robbins et al., 2021; Spang et al., 2012), which likely omits the need for GHs and CEs to break down complex sugars. Furthermore, ammonia‐oxidizing archaea do not have peptidoglycan cell walls, but an S‐layer and as such do not require enzymes for the modification of peptidoglycans during growth.

In addition to its role in catabolizing glycoconjugates and carbohydrates, the Planctomycetota (g_UBA1268) also encoded a polyphosphate kinase (*ppk*). Polyphosphate kinase catalyses the reversible transfer of the terminal phosphate of ATP to form a long‐chain polyphosphate (polyP), which can contribute to the phosphorus sequestration in *Ianthella basta* (Zhang et al., 2015). This gene was further found in the Gammaproteobacterial symbiont and the two low abundance Alphaproteobacteria (g_Ruegeria and g_UBA2767) and expressed in the metaproteome of *I. basta*.

### *Ianthella basta's* microbiome is characterized by syntrophy

Sponge symbionts do not only interact with their host, but they also have the potential to engage in complex interactions with each other. Microbes that are unable to synthesize a particular compound themselves (auxotrophy) have to live off products excreted by other species in the community (syntrophy) in order to persist in the environment. For example, in *I. basta*, all symbionts, apart from the Alphaproteobacterium (g_UBA2767), were auxotrophic for at least one of the six essential B‐vitamins, that is, thiamine (vitamin B1), riboflavin (B2), pantothenate (B5), pyridoxine (B6), biotin (B7), or cobalamin (B12). B‐vitamins are essential for the central metabolism of microorganisms (Combs Jr & McClung, 2016), including the biosynthesis of branched amino acids and methionine, and microbes that cannot synthesize these vitamins themselves must acquire them from the environment. Thiamine for example, could only be biosynthesized by the Alphaproteobacterium (g_UBA2767) and Thaumarchaeotum (Figure 2). The other symbionts could import thiamine through the thiamine transporter (*tbpA*, *thiPQ*), which was encoded and expressed in the proteomes of the three dominant symbionts and encoded in Ruegeria. All of the other essential B‐vitamins (i.e. B2, B5, B6, B7, and B12) could be biosynthesized by at least four of *I. basta*'s symbionts, leaving up to two symbionts auxotrophic to the respective vitamin (Figure 2). For example, genes necessary for cobalamin production were identified in the three alphaproteobacterial symbionts and the Thaumarchaeotum (Figure 2), which was further supported by their unique ability to produce heme, a co‐factor necessary for cobalamin production. In contrast, the Gammaproteobacterium and Planctomycetota (g_UBA1268), cobalamin auxotrophs, require an external source of cobalamin to persist in the environment (Figure 2).

### Microbial mobility and attachment

Symbionts must be able to avoid phagocytosis by host cells to maintain stable symbioses and it has been hypothesized that eukaryotic‐like proteins (ELPs), such as ankyrin (ARP), WD40, NHL, leucine‐rich (LRR), tetratricopeptide (TPR), and HEAT repeat families play an important role in this process (Jahn et al., 2019; Nguyen et al., 2014; Reynolds & Thomas, 2016). For example, ankyrin‐domain‐containing‐proteins encoded by phages could modulate the immune response of a eukaryotic host against bacteria (Jahn et al., 2019). All six symbionts in *I. basta* encoded at least one type of ELP and all ELPs were expressed in *I. basta*'s microbiome (Tables S3 and S4). However, the distribution of ELPs varied between symbionts, where the Alphaproteobacterium (g_UBA2767) encoded ARP, WD40, LRR, NHL, TPR, and HEAT repeat families (50 copies in total), displaying the most diverse array of ELPs, the dominant Alphaproteobacterium ('*Ca*. Luteria ianthellae') only encoded TPR repeat families (19 copies). The factors driving these differences and whether each ELP serves a different function in maintaining stable symbiosis remains to be elucidated.

Persistence of microbes within the sponge can also be facilitated by direct attachment to host tissue or by attachment to other microbes that are attached to the host tissue. Proteins that mediate cell–cell adhesion and biofilm formation, such as cadherin domains and fibronectin‐binding proteins, were found to be enriched in sponge microbiomes (Robbins et al., 2021) and may play similarly important roles in maintaining symbiosis in *I. basta*. Cadherin domains can bind directly to cell surfaces or to cellulose, xylan, and related compounds, mediating not only attachment to the host tissue, but also the degradation of complex carbohydrates by microbes (Fraiberg et al., 2010; Fraiberg et al., 2011). Fibronectin‐binding proteins are proteins that bind to fibronectin III domains, which can be found in both eukaryotes and prokaryotes (Hymes & Klaenhammer, 2016; Schwarz‐Linek et al., 2003). Cadherin domains were encoded in the Planctomycetota (g_UBA1268) and Alphaproteobacterium (g_UBA2767) and encoded and expressed in the Gammaproteobacterium proteome (Figure 2, Tables S3 and S4). Interestingly, these three genomes also contained genes for GH10 and CE1 that degrade xylan, a process that could be facilitated by attachment to the carbohydrate itself through cadherin domains. Genes for fibronectin‐binding proteins were identified in the Planctomycetota (g_UBA1268), the Gammaproteobacterium, and the Thaumarchaeotum. Fibronectin III domains were encoded in the Planctomycetota (g_UBA1268) and encoded and expressed in the Gammaproteobacterium, allowing for biofilm formation. No fibronectin‐binding proteins were found in the two low abundance Alphaproteobacteria (g_UBA2767 and g_Ruegeria). Interestingly, these microbes encoded a flagellum and expressed the flagellar protein (fliL; Figure 2, Tables S3 and S4), suggesting these symbionts are motile. In addition to motility, flagella can support adhesion (Kimkes & Heinemann, 2020), where fliL could play an important role in sensing the presence of a surface (Lee & Belas, 2015). Thus, the two low abundance Alphaproteobacteria may use their flagella as an alternative mechanism to maintain stable symbiosis.

The dominant Alphaproteobacterium ('*Ca*. Luteria ianthellae') lacked attachment proteins, suggesting it uses alternative mechanisms to persist within the sponge tissue. This genome instead encoded and expressed secretion system III (Figure S4, Tables S3 and S4), which can be used by microorganisms to attach to their host and inject effector proteins to modulate its metabolism (Costa et al., 2015). These proteins often contain signal peptides that mark them for secretion. In the dominant Alphaproteobacterium, signal peptides were identified on TPR repeat proteins and ubiquitin‐like proteins (i.e. proteins that can modify other proteins after translation). As TPRs and ubiquitin‐like proteins have previously been linked to symbiont establishment within the host (Robbins et al., 2021; Thomas et al., 2010; Zhou & Zhu, 2015), injection of these proteins could aid the persistence of the Alphaproteobacterium within the sponge tissue by altering *I. basta*'s metabolism (Figure S4). Additionally, secretion system III has been found to facilitate direct attachment of pathogenic bacteria to host tissue through the insertion of the so‐called virulence factor translocated intimin receptor (Tir) into the host membrane (Franzin & Sircili, 2015; Kenny et al., 1997). Once Tir is injected into the host's membrane through secretion system III, it can bind to an inverse autotransporter located in the membrane of the bacterium (Kenny et al., 1997). The dominant Alphaproteobacterium also encoded and expressed an inverse autotransporter, which would allow the symbiont to directly anchor itself to the host tissue if Tir is translocated by secretion system III (Figure S4).

To investigate whether the ability to attach to the host tissue through Tir and the inverse autotransporter was present in other sponge symbionts, we searched all available sponge symbiont genomes for secretion system III and the inverse autotransporter. Both proteins were found in three Alphaproteobacterial MAGs (GCA_014239005.1, SAMN15855071, and SAMN15855069) (Bayer et al., 2020; Robbins et al., 2021), all of which one belonged to the same order as '*Ca*. Luteria ianthellae' (o_JABSOH01), and in two MAGs belonging to the Endozoicomonadaceae family (GCA_002238585.1 and SAMN15854979) (Robbins et al., 2021; Slaby et al., 2017), suggesting this mechanism aids attachment in a variety of sponge symbionts. The ability to anchor to host tissue would indicate a tight symbiosis and consistent with such a highly dependent lifestyle, the dominant Alphaproteobacterium in *I. basta* lacked a multitude of metabolic pathways, such as the pentose phosphate cycle, vitamin production (apart from cobalamin), fatty acid B‐oxidation, aromatic ring degradation, nitrogen metabolism, and dTDP‐l‐rhamnose biosynthesis (Figure S4). It further encoded and expressed a wide range of transporters used to import essential nutrients, including amino acid transporters and sugar transporters (Figure S4). Lastly, '*Ca*. Luteria ianthellae' encoded several genes involved in DMSP metabolism, which can be a source of reduced carbon and sulfur for marine bacteria. DMSP metabolism was also identified in ‘*Ca*. Taurinisymbion ianthellae’ (Moeller et al., 2022), suggesting that DMSP, in addition to taurine, may be an important substrate for *I. basta* symbionts.

### Low abundance microbes are sources of secondary metabolite clusters

Marine sponges are rich sources of bioactive and economically important compounds that are frequently produced by their microbial symbionts and potentially aid in the chemical defence of the holobiont (Rust et al., 2020; Tianero et al., 2019; Wilson et al., 2014). *I. basta* is known to produce bastadins (Kazlauskas et al., 1981; Kunze et al., 2013; Pordesimo & Schmitz, 1990), a biotechnologically relevant secondary metabolite that can act as an antifouling agent and has cytotoxic activity against human tumour cells (Bayer et al., 2011; Greve et al., 2008). Here, we searched the genomes of the six symbionts of *I. basta* for the ability to produce bioactive compounds and found a total of 22 biosynthetic gene clusters (BGCs), of which 21 were encoded in genomes from the low abundance members of the community. (Figure 2, Table S5). Notably, genome size correlated with the number of encoded BGCs, with Ruegeria having the largest genome and most BGCs (4.33 kb, 13 BGCs), followed by UBA2767 (4.30 kb, 4 BGCs), UBA1267 (3.26 kb, 4 BGCs), '*Ca*. Taurinisymbion ianthellae' (2.08 kb, no BGCs), '*Ca*. Luteria ianthellae' (2.01 kb, no BGCs), and '*Ca*. Nitrosospongia ianthellae' (1.78 kb, 1 BGC). The most abundant BGCs were non‐ribosomal peptide synthases (NRPS/NRPS‐like, 6 clusters), hserlactones (4 clusters), and type I and III polyketide synthases (T1PKS and T3PKS, 3 clusters). Only five BGCs showed similarity to previously identified clusters, frequently with similarity scores below 30%, highlighting the novelty of the BGCs in *I. basta* (Table S5). One BGC, an NRPS‐like cluster encoded in the genome of the Planctomycetota (g_UBA1267), showed 100% similarity to a previously identified BGC thought to produce 1‐heptadecene. Interestingly, 1‐n‐heptadecane can be used by denitrifying bacteria to grow anaerobically (Spormann & Widdel, 2000), but whether this is the case for Planctomycetota or whether 1‐heptadecene is used by the other symbionts as an energy source remains to be determined. Although the novelty of *I. basta*'s BGCs precluded further identification of the metabolites produced, the identification of BGCs is the first step towards the discovery of new bioactive compounds. Thus, we suggest future research focus on identifying the activity of these BGCs.

### *Ianthella basta's* virome extends the metabolic capability of the holobiont

Marine sponges host distinct viral communities that shape their microbiome, alter host–microbe interactions (Jahn et al., 2019; Pascelli et al., 2020), and directly contribute to their host's (eukaryotic or prokaryotic) metabolism through auxiliary metabolic genes (AMGs). Viruses can further mediate horizontal gene transfer between members of the holobiont. Ankyrin repeat proteins, for example, were suggested to be horizontally transferred by viruses as these proteins are widespread in sponge symbionts from different sponge species (Reynolds & Thomas, 2016) and enriched in the sponge virome (Pascelli et al., 2020). Similarly, any of the previously described functions/proteins in *I. basta*'s microbiome can theoretically be horizontally transferred by viruses or have a viral origin.

To identify putative viral sequences in *I. basta*, VirSorter was run on all metagenomic assemblies. A total of 276 viral contigs were identified, of which 250 were phage and 25 prophage (Table S6). Of the 250 contigs that were assigned as phage, 25 were within category 1 (‘most confident’ predictions), 157 in Category 2 (‘likely’ predictions), and 68 in Category 3 (‘possible’ predictions). For the prophages, 4 were in Category 2 and 21 in Category 3 (Table S6). Of all prophages, nine were captured in bacterial MAGs and one in the Thaumarchaeotal MAG, which encoded a Type IV toxin–antitoxin system. Interestingly, toxin–antitoxin systems were previously found to be enriched in sponge associated Thaumarchaeota (Haber et al., 2021; Moeller et al., 2019). Taxonomy was subsequently assigned to all viral contigs by blasting them against the viral RefSeq Nr database and their viral origin and taxonomy was further cross‐validated by blasting them against previously recovered viral sequences from *I. basta*. The most diverse viral taxa in *I. basta* were dsDNA viruses from the order *Caudovirales*, including the families *Myoviridae*, *Podoviridae*, and *Siphoviridae* and ssDNA viruses from the order *Microviridae* (Table S6). Interestingly, we identified a *Lavidaviridae* in *I. basta*'s virome, which are known virophages that can coinfect eukaryotes with giant dsDNA viruses of the family *Mimiviridae* (Fischer, 2020). Although no contigs were assigned to the family *Mimiviridae* in this study, *Mimiviridae* were previously identified in *I. basta* and suggested to infect sponge amoeba‐like cells (Pascelli et al., 2020).

Viral contigs were functionally annotated to investigate the role of the virome in the overall metabolic capability of *I. basta*. A total of 854 genes were identified across all viral contigs, of which 89% could be functionally annotated (Table S6). Most annotated genes were replication related genes, such as transposable elements and viral DNA replication genes (Table S6). However, putative auxiliary metabolic genes (AMGs) were also detected (Figure 2, Table S6). For example, eukaryotic‐like proteins, such as TPR repeat proteins and WD40 repeat proteins, were found on seven viral contigs, of which two were captured in the MAGs of the Thaumarchaeotum (prophage, Category 3) and Alphaproteobacterium (g_Ruegeria; prophage, Category 2). Interestingly, TPR repeat proteins were previously suggested to be laterally transferred between sponge symbionts (Robbins et al., 2021), which could be mediated by viruses. Similarly, attachment proteins, including cadherins, are thought to be laterally transferred between sponge symbionts and were identified on the viral contigs in *I. basta* (Figure 2, Table S6). *I. basta*'s virome also encoded various transporters, such as the spermidine/putrescine transport system (prophage Category 2, binned in the Alphaproteobacterium from the genus Ruegeria). Spermidine and putrescine are polyamines that play essential roles in cell differentiation and proliferation. Thus, the virus‐encoded spermidine/putrescine transport system could further aid in the overall transport of vital compounds in *I. basta*'s microbiome.

## CONCLUSION AND OUTLOOK

To fully understand the functioning of the sponge holobiont and establish a model species for future experiments, we present a metaproteogenomic analysis of >90% of *I. basta*'s microbiome and virome, providing key insights into the unique functional role of each symbiont and their metabolic interplay. We show that low abundance microbes drive fundamentally different processes compared with the dominant symbionts (e.g. complex carbohydrate degradation and vitamin production) and are repositories of key functions involved in nitrogen cycling and secondary metabolite production. We further highlight the auxotrophic nature of sponge symbionts to vitamin production and their dependency on other microbes in the environment. *Ianthella basta*'s symbionts were also predicted to use a variety of mechanisms to avoid host phagocytosis and to attach to the host tissue. These mechanisms, such as the presence of secretion system III, could be further investigated using cryo‐EM. Lastly, we characterized *I. basta*'s virome by identifying putative viral contigs in its microbiome, cross‐validating each sequence against previously recovered viral sequences from the sponge. Functional characterization of *I. basta*'s virome suggested a role for viruses in the lateral gene transfer of ELPs and attachment proteins and their ability to extend the metabolic capability of the holobiont through transporters. Taken together, this metaproteogenomic data provide the metabolic framework to adopt *I. basta* as a model organism for studying host–microbe interactions, particularly the establishment, development, and maintenance of sponge symbionts.

## AUTHOR CONTRIBUTIONS

**Pamela Engelberts:** Conceptualization (lead); data curation (lead); formal analysis (lead); investigation (lead); methodology (lead); validation (lead); visualization (lead); writing – original draft (lead); writing – review and editing (equal). **Steven Robbins:** Data curation (supporting); formal analysis (supporting); investigation (supporting); methodology (supporting); supervision (lead); validation (supporting); writing – review and editing (supporting). **Craig W Herbold:** Formal analysis (equal); investigation (equal); methodology (equal); writing – review and editing (supporting). **Florian Moeller:** Investigation (supporting); validation (supporting). **Nico Jehmlich:** Data curation (supporting); formal analysis (supporting); investigation (equal); methodology (supporting); writing – review and editing (supporting). **Patrick Laffy:** Formal analysis (supporting); investigation (supporting); writing – review and editing (supporting). **Michael Wagner:** Resources (equal); supervision (supporting); writing – review and editing (equal). **Nicole Webster:** Resources (equal); supervision (supporting); writing – review and editing (supporting).

## CONFLICT OF INTEREST

The authors declare no conflict of interest.

## ETHICAL STATEMENT

All prevailing scientific ethical practices have been respected.

## Supporting information


**Figure S1.** Phylogenomic tree based on 564 publicly available Thaumarchaeotal MAGs, showing the placement of the Thaumarchaeotal MAG from *I. basta*. The outgroup consists of MAGs belonging to the phylum Undinarchaeota. The tree was clustered on orders, only showing the closest relatives to the recovered MAG from this study (displayed in red) and their sponge hosts.

**Figure S2.** Phylogenomic tree based on 8980 publicly available Gammaproteobacterial MAGs and two Gammaproteobacterial MAGs recovered from *Hexadella detritifera* in this study, showing the placement of the Gammaproteobacterial MAG from *I. basta*. The outgroup consists of MAGs belonging to the phylum Chloroflexota. The tree was clustered on orders and families, only showing the closest relatives to the recovered MAG from this study (displayed in red) and their sponge hosts.

**Figure S3.** Phylogenomic tree based on 7361 publicly available Alphaproteobacterial MAGs and one Alphaproteobacterial MAG recovered from *Hexadella detritifera* in this study, showing the placement of the Alphaproteobacterial MAG from *I. basta*. The outgroup consists of MAGs belonging to the phylum Chloroflexota. The tree was clustered on orders, only showing the closest relatives to the recovered MAG from this study (displayed in red) and their sponge hosts.

**Figure S4.** Metabolic reconstruction of the dominant Alphaproteobacterium in *Ianthella basta* ('*Ca*. Luteria ianthellae'). Genes encoded in the genome are displayed in black, genes absent from the genome are red, and genes expressed in the metaproteomics dataset are blue. The Alphaproteobacterium lacks the pentose phosphate pathway, pathways to metabolize vitamins, fatty acid B‐oxidation, aromatic ring degradation, dTDP‐l‐rhamnose biosynthesis, and secondary metabolite clusters. The metabolic reconstruction of the dominant Thaumarchaeotum and Gammaproteobacterium can be found in Moeller et al. (2019) and Moeller et al. (2022), respectively.

**Table S1.** Taxonomy and statistics of all 43 MAGs retrieved from the four *Ianthella basta* individuals. The six MAGs used for metabolic reconstruction are highlighted in blue. Taxonomy as per GTDB taxonomy Release 202 is shown.

**Table S2.** Percentage identity between 16S rRNA gene amplicon sequences of the three dominant symbionts of the yellow (thin and thick, unpublished data) and purple (thick; Engelberts et al., n.d.) morphotypes and the genomes retrieved from this study (yellow, thin).

**Table S3.** Expressed proteins in the four *Ianthella basta* individuals. Each protein sequence was given a unique sequence ID (seqID). If the sequence was found to be part of another sequence (i.e. redundant), the ‘unique’ column received a value of ‘0’, with the ‘representative seqID’ column displaying the representative sequence. The columns ‘redundantGeneID’ and ‘uniqueGeneID’ are a numerical index. All seqIDs with the same redundantGeneID come from the same scaffold with the same coordinates and orientation. All SeqIDs with the same ‘uniqueGeneID’ have the exact same peptide sequence predicted regardless of which dataset/scaffold it comes from. Information on the source assembly, whether the protein was captured in a MAG, and the functional of the sequence are also included in the table.

**Table S4.** KO count for the six MAGs retrieved from the four *Ianthella basta* individuals used for metabolic reconstruction. MAG IDs consist of the MAG ID from Table S1 combined with the lowest resolved taxonomy as per GTDB taxonomy Release 202.

**Table S5.** Secondary metabolite clusters identified in each of the six MAGs retrieved from *Ianthella basta* used for metabolic reconstruction. MAG IDs consist of the MAG ID from Table S1 combined with the lowest resolved taxonomy as per GTDB taxonomy Release 202.

**Table S6.** Taxonomy and function of all putative viral contigs in the metagenomic assemblies of *Ianthella basta*. Metagenomic assemblies containing the codes H1, H2, or M1‐M4 originated from the six sequencing runs on *Ianthella basta* from Moeller et al. (2019). For metagenomic assemblies from this study: IB stands for *Ianthella basta* followed by the replicate number and whether the individual was deeply or shallowly sequenced. Taxonomy was included for each viral contig based on its match against previously recovered *I. basta* viral sequences and the viral component of the RefSeq NR database. Further information included in the table is whether the viral contig was binned in one of the symbiont genomes, whether the contig was phage or prophage and to what category it belonged. Viral contigs that contained multiple gene annotations are highlighted in pink.

**Supplementary Note 1:** Supporting Information

## Data Availability

Raw reads and MAGs are available at NCBI under Bioproject ID PRJNA807825. The mass spectrometry proteomics data have been deposited to the ProteomeXchange Consortium via the PRIDE (Perez‐Riverol et al., 2022) partner repository with the dataset identifier PXD032278.
