# Supplementary figures and images for: Metabolic reconstruction of the near complete microbiome of the model sponge Ianthella basta
Source: Environ Microbiol. 2022 Dec 23;25(3):646–60. doi: 10.1111/1462-2920.16302 (PMC10947273; doi:10.1111/1462-2920.16302)

Tree scale: 1

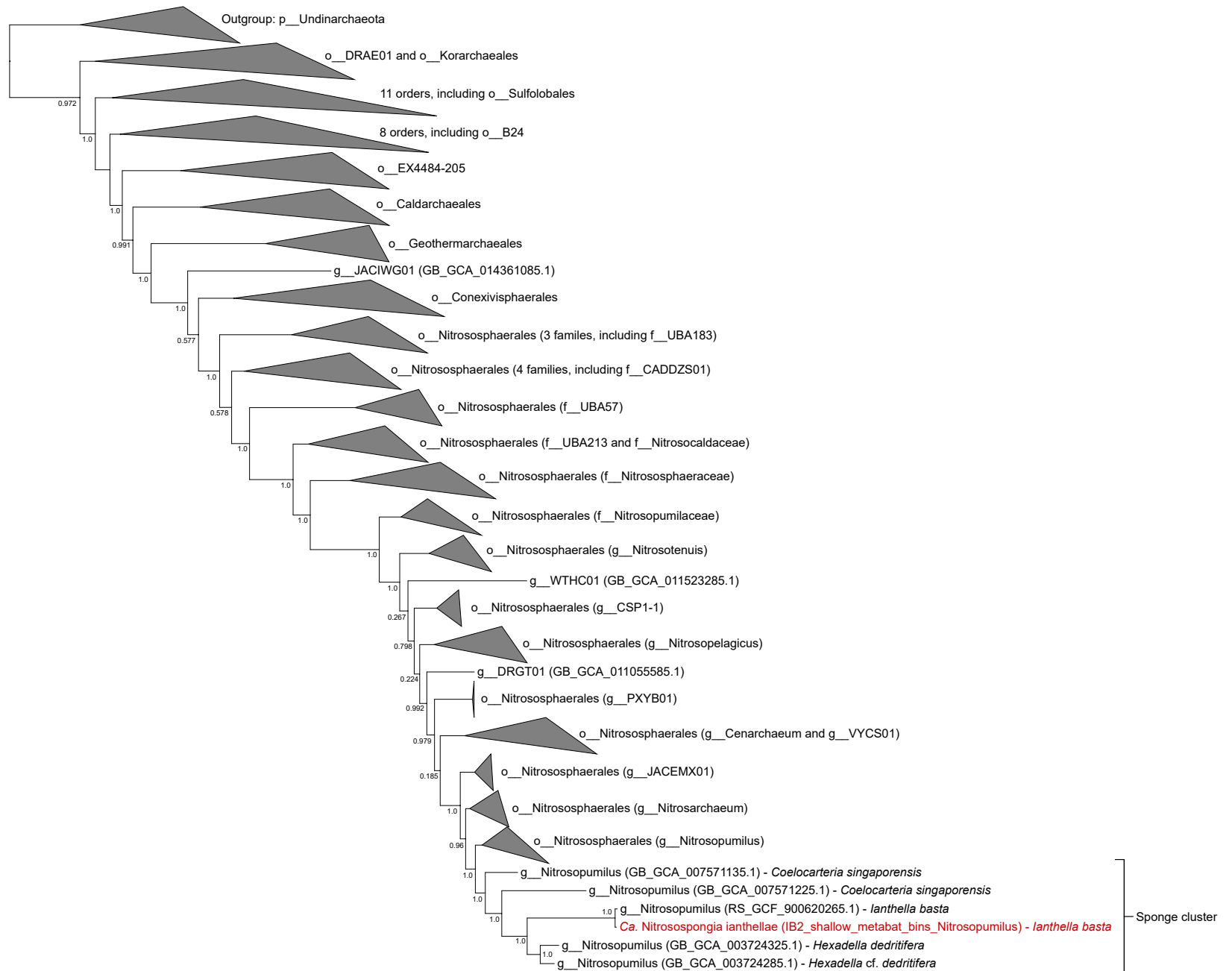

Supplement: Supplementary file 1 — Figure S1. Phylogenomic tree based on 564 publicly available Thaumarchaeotal MAGs, showing the placement of the Thaumarchaeotal MAG from I. basta. The outgroup consists of MAGs belonging to the phylum Undinarchaeota. The tree was clustered on orders, only showing the closest relatives to the recovered MAG from this study (displayed in red) and their sponge hosts. [file EMI-25-646-s001.pdf]

Tree scale: 1

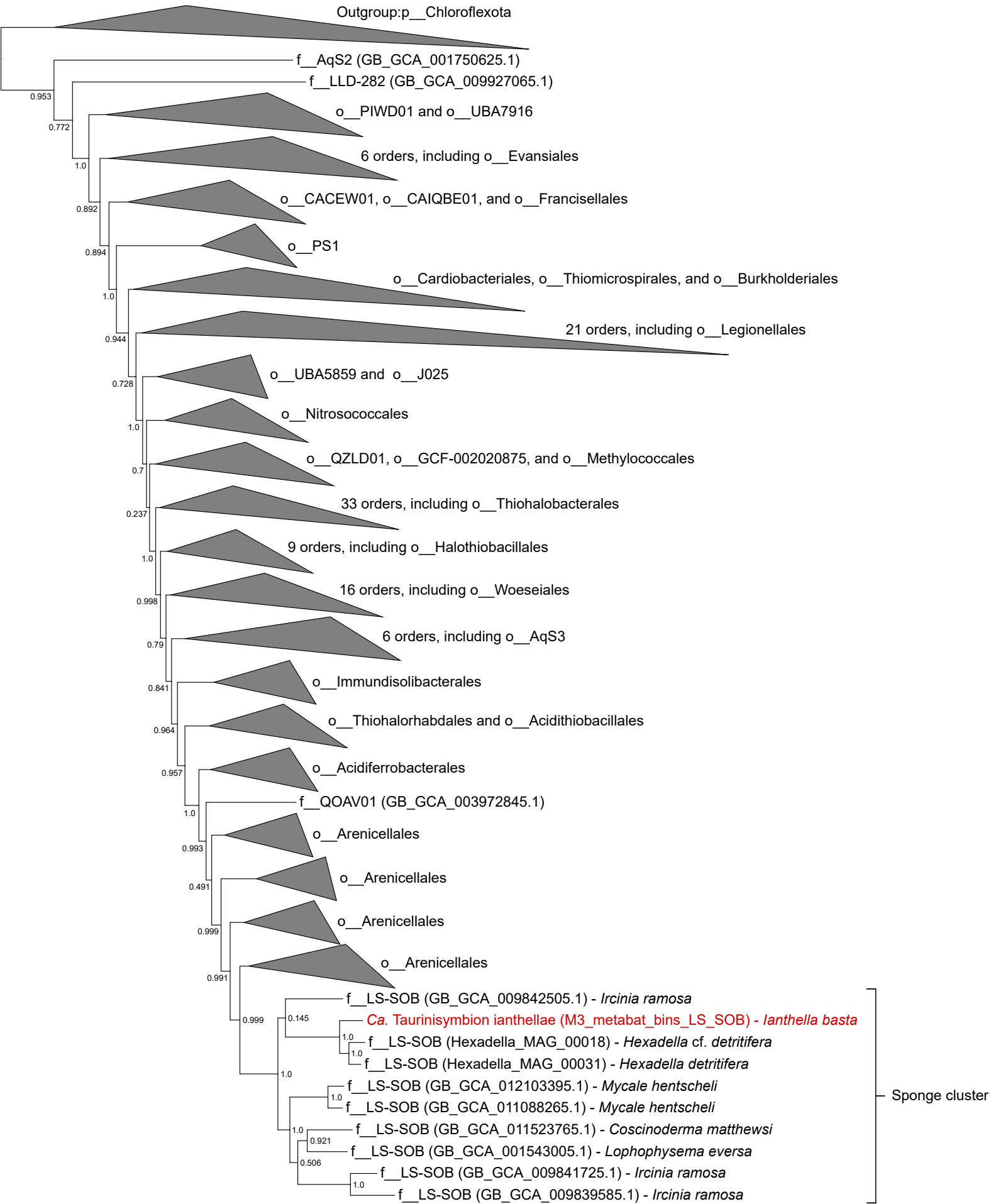

Supplement: Supplementary file 2 — Figure S2. Phylogenomic tree based on 8980 publicly available Gammaproteobacterial MAGs and two Gammaproteobacterial MAGs recovered from Hexadella detritifera in this study, showing the placement of the Gammaproteobacterial MAG from I. basta. The outgroup consists of MAGs belonging to the phylum Chloroflexota. The tree was clustered on orders and families, only showing the closest relatives to the recovered MAG from this study (displayed in red) and their sponge hosts. [file EMI-25-646-s005.pdf]

Tree scale: 1

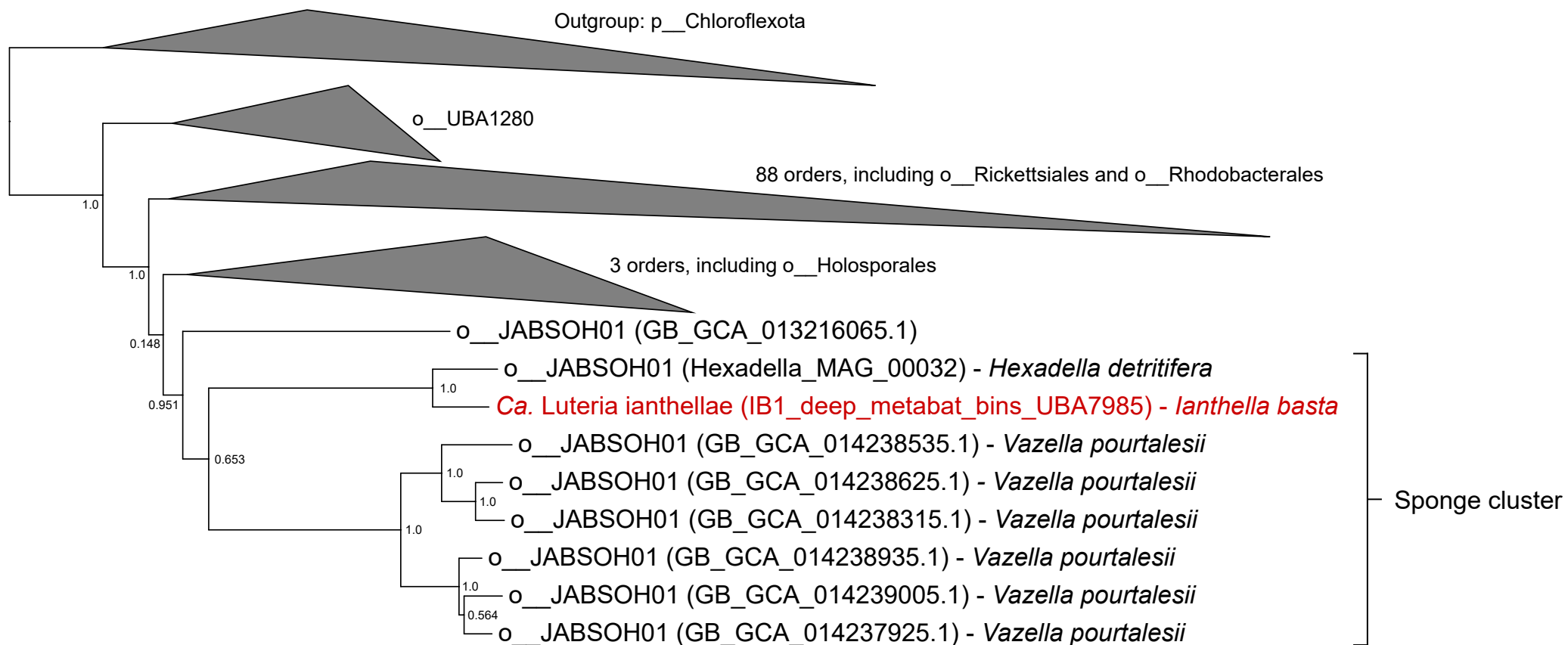

Supplement: Supplementary file 3 — Figure S3. Phylogenomic tree based on 7361 publicly available Alphaproteobacterial MAGs and one Alphaproteobacterial MAG recovered from Hexadella detritifera in this study, showing the placement of the Alphaproteobacterial MAG from I. basta. The outgroup consists of MAGs belonging to the phylum Chloroflexota. The tree was clustered on orders, only showing the closest relatives to the recovered MAG from this study (displayed in red) and their sponge hosts. [file EMI-25-646-s002.pdf]

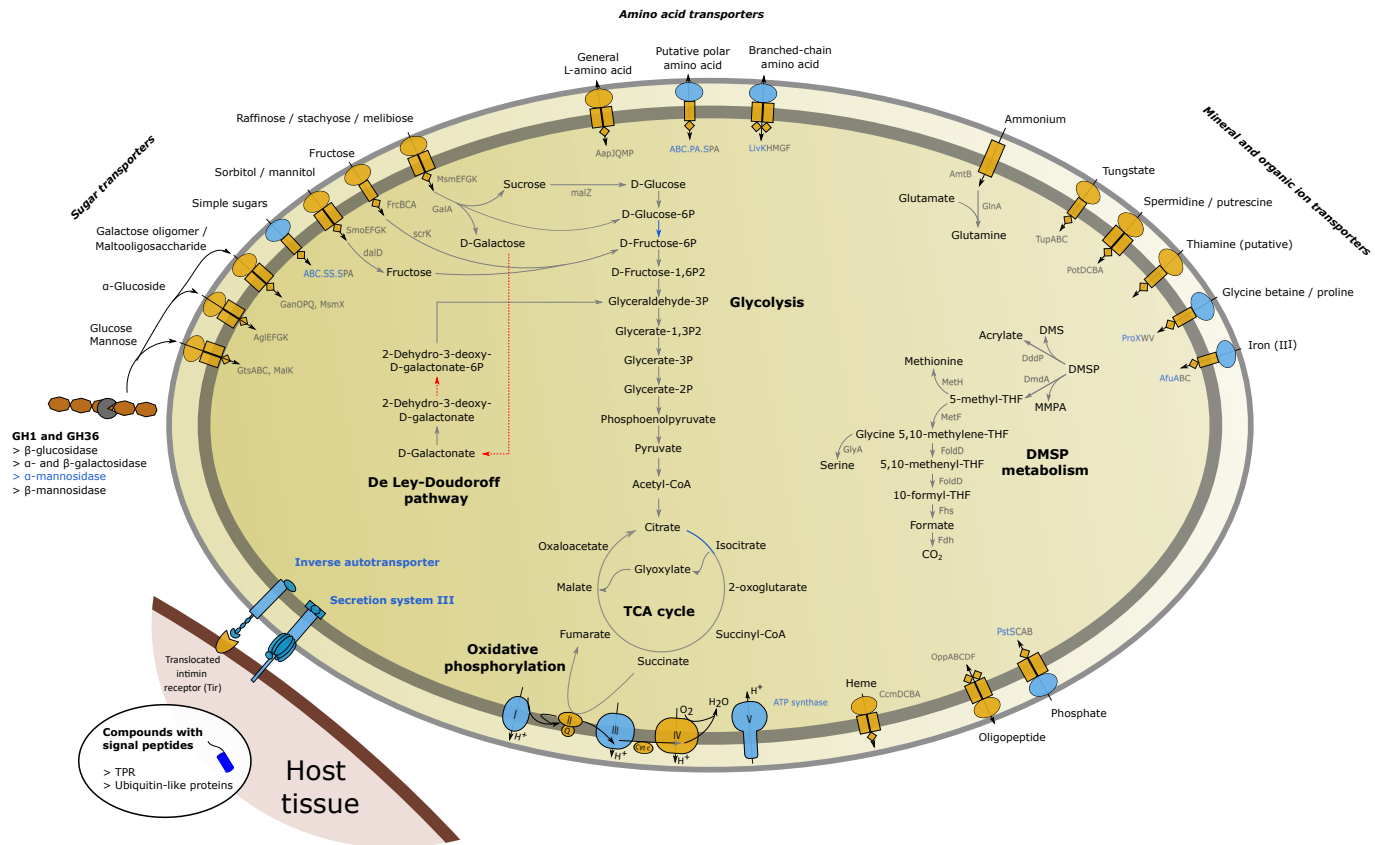

Supplement: Supplementary file 4 — Figure S4. Metabolic reconstruction of the dominant Alphaproteobacterium in Ianthella basta ('Ca. Luteria ianthellae'). Genes encoded in the genome are displayed in black, genes absent from the genome are red, and genes expressed in the metaproteomics dataset are blue. The Alphaproteobacterium lacks the pentose phosphate pathway, pathways to metabolize vitamins, fatty acid B‐oxidation, aromatic ring degradation, dTDP‐l‐rhamnose biosynthesis, and secondary metabolite clusters. The metabolic reconstruction of the dominant Thaumarchaeotum and Gammaproteobacterium can be found in Moeller et al. (2019) and Moeller et al. (2022), respectively. [file EMI-25-646-s011.pdf]
