# Supplementary material for: Metabolic reconstruction of the near complete microbiome of the model sponge Ianthella basta
Source: Environ Microbiol. 2022 Dec 23;25(3):646–60. doi: 10.1111/1462-2920.16302 (PMC10947273; doi:10.1111/1462-2920.16302)
Supplement: Supplementary file 11 — Supplementary Note 1: Supporting Information [file EMI-25-646-s008.docx]

**Supplementary Information**

Metabolic reconstruction of the near complete microbiome of the model sponge *Ianthella basta*

J. Pamela Engelberts, Steven J. Robbins, Craig W. Herbold, Florian U. Moeller, Nico Jehmlich, Patrick W. Laffy, Michael Wagner, and Nicole S. Webster

**Supplementary Note 1**

To recover microbial genomes from the sponge *Hexadella detritifera*, paired end reads from SRA runs SRR8088660, SRR8088661, SRR8088662, and SRR8088663 were downloaded and adapters and residual phiX were removed using BBduk (BBMap v37.61) [1]. Reads were then quality filtered (minlen=99 and trimq=15), denoised using BayesHammer (SPAdes v3.15.3) [2], and merged using bbmerge (BBMap v37.61) [1]. Reads were subsequently assembled using SPAdes v3.15.3 [2] and metaSPAdes v3.14.0 [3], which resulted in eight assemblies (four per assembler). Binning of all data was performed using MetaBAT v1 [4] and MetaBAT v2.15 [5]. MAGs from each SRA run were dereplicated at 99% identity using Drep v.1.4.3 [6]. “Winning” MAGs from each SRA run were then combined and dereplicated at 96.5% identity using dRep v1.4.3 [6]. To assign taxonomy to each MAG, GTDB-Tk v1.5.0 [7], which is based on the Genome Taxonomy Database (GTDB, <http://gtdb.ecogenomic.org>) taxonomy Release 202, was used, which resulted in one MAG belonging to the Alphaproteobacterial order JABSOH01 and two Gammaproteobacterial MAGs belonging to the family LS-SOB. These three genomes have been submitted to NCBI under Bioproject ID PRJNA807825.

**References**

1. Bushnell, B., *BBMap: a fast, accurate, splice-aware aligner*. 2014, Lawrence Berkeley National Lab.(LBNL), Berkeley, CA (United States).

2. Bankevich, A., et al., *SPAdes: a new genome assembly algorithm and its applications to single-cell sequencing.* J Comput Biol, 2012. **19**(5): p. 455-77.

3. Nurk, S., et al., *metaSPAdes: a new versatile metagenomic assembler.* Genome Res, 2017. **27**(5): p. 824-834.

4. Kang, D.D., et al., *MetaBAT, an efficient tool for accurately reconstructing single genomes from complex microbial communities.* PeerJ, 2015. **3**: p. e1165.

5. Kang, D.D., et al., *MetaBAT 2: an adaptive binning algorithm for robust and efficient genome reconstruction from metagenome assemblies.* PeerJ, 2019. **7**: p. e7359.

6. Olm, M.R., et al., *dRep: a tool for fast and accurate genomic comparisons that enables improved genome recovery from metagenomes through de-replication.* ISME J, 2017. **11**(12): p. 2864-2868.

7. Chaumeil, P.-A., et al., *GTDB-Tk: a toolkit to classify genomes with the Genome Taxonomy Database.* Bioinformatics, 2019. **36**(6): p. 1925-1927.
